# The Influence of Content Meaningfulness on Eye Movements across Tasks: Evidence from Scene Viewing and Reading

**DOI:** 10.3389/fpsyg.2016.00257

**Published:** 2016-03-01

**Authors:** Steven G. Luke, John M. Henderson

**Affiliations:** ^1^Department of Psychology and Neuroscience Center, Brigham Young University, ProvoUT, USA; ^2^Department of Psychology, University of California Davis, DavisCA, USA; ^3^Center for Mind and Brain, University of California, Davis, DavisCA, USA

**Keywords:** eye movements, cognitive control, meaning, reading, scene perception, eye tracking, eye movement control

## Abstract

The present study investigated the influence of content meaningfulness on eye-movement control in reading and scene viewing. Texts and scenes were manipulated to make them uninterpretable, and then eye-movements in reading and scene-viewing were compared to those in pseudo-reading and pseudo-scene viewing. Fixation durations and saccade amplitudes were greater for pseudo-stimuli. The effect of the removal of meaning was seen exclusively in the tail of the fixation duration distribution in both tasks, and the size of this effect was the same across tasks. These findings suggest that eye movements are controlled by a common mechanism in reading and scene viewing. They also indicate that not all eye movements are responsive to the meaningfulness of stimulus content. Implications for models of eye movement control are discussed.

## Introduction

When we are performing a visual task, such as reading, searching for an object, or just looking around at the world, our eyes make rapid movements, called *saccades*, several times a second. In between these saccades, the eyes make pauses (called *fixations*) to take in visual information. Researchers who study eye movements are interested in why we look where we do and why we move our eyes when we do. It is thought by many that eye movements are under cognitive control, meaning that the where and when of eye movements are influenced by cognitive processes related to perception, memory, and language (Rayner, 2009; Rayner and Reingold, 2015; but see Yang and McConkie, 2001; Vitu, 2003 for an alternate viewpoint). In short, cognitive control implies that our eye movements respond quickly to the nature of what we are looking at in any given moment.

The study of reading has proven to be fertile ground for investigating the influence of cognitive factors on eye movement control. This is primarily because words have multiple quantifiable cognitive properties such as frequency and predictability, properties that influence the processing of those words in multiple ways. Studies exploring the influence of these cognitive properties have shown that cognitive processing can influence eye movements on a moment-by-moment basis (Just and Carpenter, 1980; Rayner, 1998, 2009; Kliegl et al., 2006).

Studying the influence of cognitive processing in other visual tasks, such as scene viewing, has proven more difficult, because the units of processing (i.e., objects) are not so clearly differentiated in visual scenes (they overlap) and their cognitive properties are not so easily defined or quantified. As a result, the influence of cognitive factors in scene viewing is less clearly understood (Henderson et al., 1999; Henderson, 2003; Võ and Henderson, 2009; Wang et al., 2010). Researchers have therefore been forced to rely more on manipulations of global image properties in order to investigate cognitive control of eye movements in these scene viewing tasks. These global manipulations, which are described below, reduce the possibility of cognitive control by removing or denying access to information needed to interpret and understand the image.

These global image manipulations are also useful because they can also be applied to reading, permitting direct cross-task comparisons. This is important because reading is measurably different from other visual tasks, such as scene viewing. For example, fixation durations are significantly shorter in reading (Henderson and Hollingworth, 1998; Rayner, 2009; Luke et al., 2013). Saccade amplitudes tend to be shorter in reading as well (Rayner, 2009; Henderson and Luke, 2014). On the other hand, in reading the eyes are presumably guided by the same neural systems as in other visual tasks, and eye movements are made for the purpose of gathering information regardless of task. In support of this is the observation that aggregate measures of eye movement behavior correlate across tasks (Henderson and Luke, 2014), although not all researchers have found this relationship with regard to reading (Rayner et al., 2007). At the same time, while many core visual and eye movement control areas appear to be common to both tasks (Choi and Henderson, 2015), cognitive control in reading and scene viewing could be exerted by different cortical centers. For example, the parahippocampal place area (PPA; Epstein and Kanwisher, 1998; Epstein et al., 1999) appears to be involved in scene viewing but not in reading (Choi and Henderson, 2015; Henderson and Choi, 2015), while the visual word form area (VWFA; Cohen et al., 2000; McCandliss et al., 2003) and language areas in the left hemisphere contribute to eye movement control in reading but not in scene viewing (Choi and Henderson, 2015; Henderson et al., 2015). So, the underlying processes that control eye movements, making them sensitive to the meaning of words and objects, might differ in significant ways in reading compared to other tasks. Identifying which processes are common to all tasks and which are task-specific is an important goal of eye-movement research.

Usually, the global image manipulations used to study eye movement control involve obscuring or removing the stimulus for an extended period of time. Sometimes participants are given a brief view of part of the text or of the scene before it is removed from view (Rayner et al., 2003, 2009). The results of these studies suggest that saccades are under cognitive control in both tasks. Another technique, called the stimulus onset delay (SOD) paradigm, involves covering all or part of the visual stimulus with a mask during predefined saccades, so that when the next fixation begins the stimulus is not visible. The mask remains on screen for a predetermined and varied delay and is then removed so that the stimulus again becomes visible. The SOD paradigm thus simulates processing difficulty in a manner that is precisely controlled and easily applied to a variety of different visual tasks. This paradigm has been employed to study eye movement control in both reading (Rayner and Pollatsek, 1981; Morrison, 1984; Dambacher et al., 2013) and scene processing (Henderson and Pierce, 2008; Henderson and Smith, 2009). The SOD paradigm has also been used to compare reading and scene viewing directly (Nuthmann and Henderson, 2012; Luke et al., 2013), and equivalent effects of delay duration on fixation durations were observed in both tasks, strongly suggesting that reading and scene viewing share a common mechanism for the control of fixation duration.

The SOD paradigm typically reveals two populations of fixations: some that outlast the delay and others that do not (Henderson and Pierce, 2008; Henderson and Smith, 2009; Luke et al., 2013). The first population of fixations appear to be under cognitive control, increasing linearly with the duration of the delay, while the second population of fixations does not lengthen in response to the presence of the mask, suggesting that these fixations are not under cognitive control. Thus, direct comparisons of eye movement control in different tasks are important not only because they tell us that these tasks share a common mechanism, but also because they reveal something about the nature of that mechanism. In some models, such as E–Z reader (Reichle et al., 1998), most or all reading saccades are initiated by successful completion of some stage of lexical access, while other models such as SWIFT (Engbert et al., 2002, 2005), CRISP (Nuthmann et al., 2010; Nuthmann and Henderson, 2012), and the competition-interaction model (Yang and McConkie, 2001) delay some saccades when cognitive processing difficulty is encountered. The existence of two different populations of fixations is more consistent with the latter class of models. Exploring eye movement control across multiple tasks can help to further adjudicate between these different proposals.

Another global method for exploring eye movement control that has been employed exclusively in reading is the pseudo-reading paradigm, in which all letters in a text are replaced with block shapes or a single letter such as Z. This manipulation removes all meaning from the text, but preserves the visual-spatial layout of the words, sentences, and paragraphs. A finding from this technique is that fixations are typically longer in pseudo-reading than in reading. This finding seems rather paradoxical at first, as one might expect longer fixations when cognitive processing is engaged, not when it is absent, as no processing difficulty should occur when there is nothing to process; nevertheless this finding has been consistently shown across studies (Vitu et al., 1995; Rayner and Fischer, 1996). If we define processing difficulty simply as an inability to identify a stimulus, such as a word, then it makes sense that fixation durations should be lengthened when meaning is removed and identification is not possible. Or it could be that having meaning facilitates processing, thereby shortening fixations relative to the meaningless text condition (Reichle et al., 2012).

Like the SOD paradigm, the pseudo-reading technique has also shown that some eye movement behaviors in reading are under the influence of cognitive control, while others appear not to be (Nuthmann et al., 2007; Henderson and Luke, 2012; Luke and Henderson, 2013). The pseudo-reading technique has also been used in combination with EEG or MRI to explore the neural bases of cognitive control in reading (Henderson et al., 2013, 2014a, 2015).

The present study uses the principle behind the pseudo-reading paradigm, the removal of meaningfulness, to directly compare the cognitive control of eye movements in reading and in scene viewing. We manipulated text and scenes to create a pseudo-reading and a pseudo-scene viewing condition, and we then compared participants’ eye movements in the two tasks and in their pseudo-variants in a within-subjects design. Based on previous research on reading, we expected increased fixation durations for meaningless stimuli (Vitu et al., 1995; Rayner and Fischer, 1996; Luke and Henderson, 2013). Filtering scenes to remove high-frequency visual information, which makes object identification difficult, has also been shown to increase fixation durations (Mannan et al., 1997; Henderson et al., 2014b). We predict, therefore, that fixation durations will be longer for our pseudo-stimuli, indicating that at least some fixations are under cognitive control.

**FIGURE 1:**
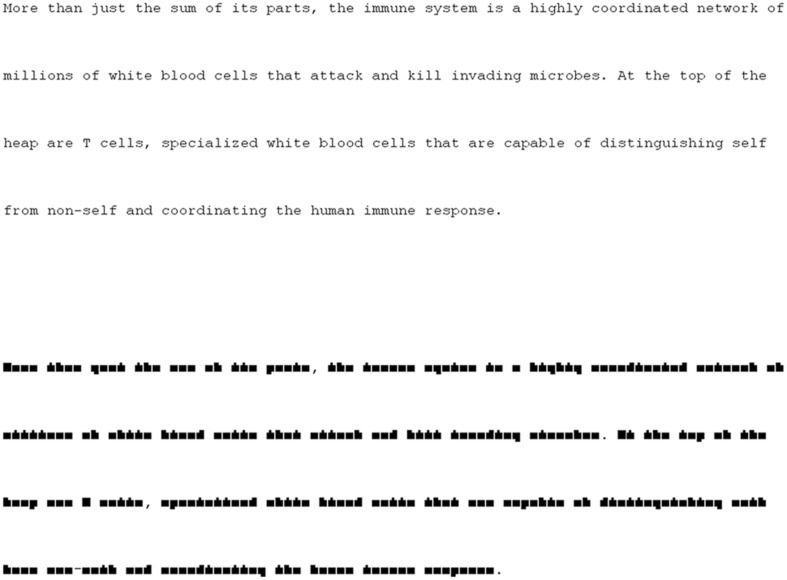
**Examples of text and pseudo-text.** Normal text is above, with the corresponding pseudo-text below.

**FIGURE 2:**
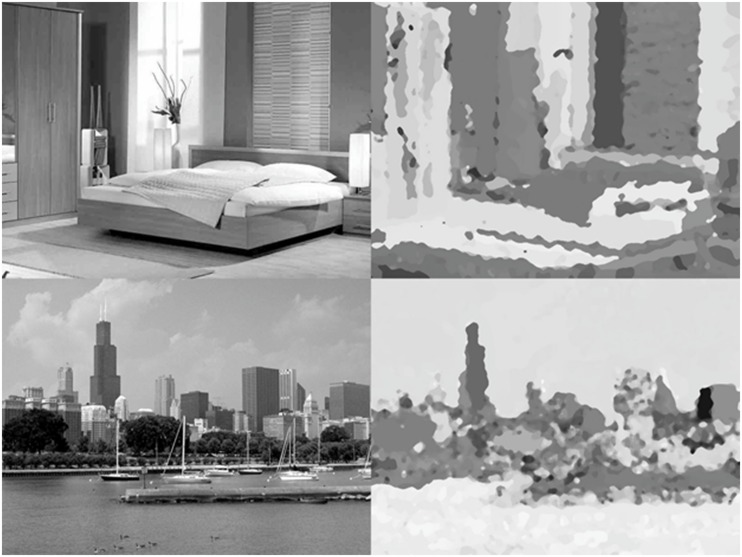
**Examples of the scene and pseudo-scene stimuli.** Normal scenes are on the left, with the corresponding pseudo-scenes on the right.

This manipulation permits us to test two more specific hypotheses about eye-movement control as well. The first is that the same systems control eye movements in reading and in scene viewing: If eye movements are controlled by the same systems across tasks, then eye movements should be influenced similarly by the removal of meaningfulness in both tasks. We note here that we use the term ‘meaningfulness’ because of the global nature of the manipulation; the pseudo-stimuli differ from the original text and scenes on many levels, but what is important is that they are not interpretable, meaning that cognitive control has little opportunity to influence eye movements for these stimuli (see **Figures 1** and **2** below). The second hypothesis is that, consistent with findings from the SOD paradigm and from pseudo-reading that not all fixations are under cognitive control (Henderson and Pierce, 2008; Henderson and Smith, 2009; Luke and Henderson, 2013; Luke et al., 2013; Henderson and Luke, 2014), the removal of meaningfulness will only affect some, and, not all, fixations.

## Materials and Methods

### Participants

Forty participants recruited from Brigham Young University completed the experiment. All participants were native English speakers with 20/20 corrected or uncorrected vision. Prior to participant recruitment, the institutional review board that Brigham Young University approved the study.

### Apparatus

Eye movements were recorded via an SR Research Eyelink 1000 plus tower mount eye tracker (spatial resolution of 0.01°) sampling at 1000 Hz. Subjects were seated 60 cm away from a 24″ LCD monitor with display resolution set to 1600 × 900, so that approximately three characters subtended 1° of visual angle. Scenes (800 × 600 pixel images) subtended 21 by 16° of visual angle. Head movements were minimized with a chin and head rest. Although viewing was binocular, eye movements were recorded from the right eye. The experiment was controlled with SR Research Experiment Builder software.

### Materials

Fifty-six short paragraphs (40–60 words) were taken from online news articles. These texts were used in Luke and Henderson (2013) and included a total of 1415 unique words: three one-letter words, 30 two-letter words, 93 three-letter words, 197 four-letter words, 247 five-letter words, 227 six-letter words, 218 seven-letter words, 159 eight-letter words, 110 nine-letter words, and 131 words 10 letters or longer. Two different versions of each text were created, a Normal Reading version, in which the text appeared on the screen in Courier New 16pt font, and a Mindless Reading version, in which the text was displayed in a custom font (also 16pt). This font transformed letters into block shapes (see **Figure 1**) while preserving overall word shape. Both fonts were monospace, and all letters, words, and lines of text appeared in exactly the same location regardless of font.

For the scene stimuli, pseudo-scenes were created that were analogous to the pseudo-texts in that they had a complex visual structure similar to that of their real scene counterparts but were not meaningful. These pseudo-scenes did not contain any identifiable objects and were not easily assigned to a particular scene category. In order to create a set of pseudo-scenes, we began with a large set of 840 images. The images depicted scenes from seven different categories, five outdoor and two indoor. The outdoor scenes were images of beaches, forests, mountains, cityscapes, and highways (Walther et al., 2009), while the indoor scenes were images of bedrooms and kitchens.

Our goal was to find a manipulation for scenes that was similar to the manipulation that we and others had previously used for text (see **Figure 1**), which still looked like text but was not interpretable. Specifically, we wanted to (1) preserve the spatial layout of the scene as much as possible, while (2) removing meaning. This proved to be a difficult task for visual scenes, and we tried and rejected several different methods. The manipulation we ultimately chose removed the meaning from these images via an extensive filtering process that extracted the edges from the images, expanded and distorted these edges, and then filled the empty areas within these newly warped edges with color, a process which also erased the edge lines. Then both the scenes and pseudo-scenes were transformed to grayscale.

This manipulation disguised the identities of the objects in the image and made the scene categories difficult to identify. The pseudo-scenes were then normed to see which ones were the most difficult to identify. All 840 meaningless images were posted on Amazon’s Mechanical Turk (Buhrmester et al., 2011). Participants were told that each image was created by altering a photograph of a scene, and asked to provide a short label identifying the category of the scene that the image was created from (or “Don’t Know” if they were unable to identify it) and to rate their confidence in the label they had provided. Each image was labeled by ten different participants.

Based on these norming data, 56 pseudo-scenes were selected, eight from each scene category. Overall, participants in the norming study gave the “Don’t Know” response for 73% of the images, and for the minority of images that they did attempt to provide a label for, they provided a correct label only 10% of the time. Confidence ratings were very low (*M* = 1.34 on a 5-point scale). Therefore, these images were extremely difficult to identify, even for participants who knew that the images had been derived from actual scenes. Examples of the images used can be seen in **Figure 2**.

### Procedure

For the reading task, participants were told that they would be reading short texts on a computer screen while their eye movements were recorded. Participants were also told that some of the texts would appear with blocks in place of letters, and that in those cases they should move their eyes as if they were reading. These are the standard instructions given in pseudo-reading experiments (Vitu et al., 1995; Rayner and Fischer, 1996; Nuthmann et al., 2007; Luke and Henderson, 2013). Participants were informed that their memory for the texts and pseudo-texts would be tested at the end of the experimental session. Each trial involved the following sequence. The trial began with a gaze trigger, a black circle presented in the position of the first character in the text. Once a stable fixation was detected on the gaze trigger, the text was presented. The participant read the text and pressed a button when finished. Then a new gaze trigger appeared and the next trial began.

For the scene task, participants were told that they would be viewing both photographs and patterns of blobs and shapes on the screen as their eye movements were monitored. Participants were further told that they should view each image in preparation for a memory test that would be administered at the end of the experiment. Each trial involved the following sequence. Each trial began with a gaze trigger, which consisted of a black circle presented in the center of the screen. Once a stable fixation had been detected on the gaze trigger, the image was presented for 10 s. At the end of 10 s, a new gaze trigger appeared and the next trial began.

Stimulus condition (Meaningful vs. Pseudo-Stimulus) was counterbalanced across two stimulus lists, separately for each task (Reading vs. Scene Viewing), and each participant saw only one of the lists. Thus, each participant saw 28 normal texts, 28 pseudo-texts, 28 normal scenes, and 28 pseudo-scenes, and no participant saw the same text or scene twice. The order of stimulus presentation was counterbalanced across participants, with half of the participants completing the scene viewing task first, and half the reading task. Within each task, stimuli were presented in a random order for each participant. For the memory test, participants were presented with a random selection of novel and previously viewed texts, scenes, and pseudo-scenes, and asked to indicate via button-press if they had seen the stimulus before. The memory test was administered solely to ensure that participants attended carefully to the experimental tasks, and so the data from the memory task were not analyzed.

## Results

Fixations shorter than 50 ms or longer than 1200 ms were removed as outliers, and saccades larger than 22° were removed to exclude return sweeps in reading. Summary statistics for all dependent variables can be found in **Table 1**.

**Table 1:** Means (and standard deviations) for the dependent variables.

	Reading		Scene viewing	
	
	Meaningful	Pseudo-text	Meaningful	Pseudo-scenes
Fixation duration	206 (89)	255 (126)	284 (143)	307 (169)
Saccade amplitude	3.22 (2.09)	3.66 (2.92)	4.26 (3.47)	4.36 (3.55)


### Saccade Amplitude

**FIGURE 3:**
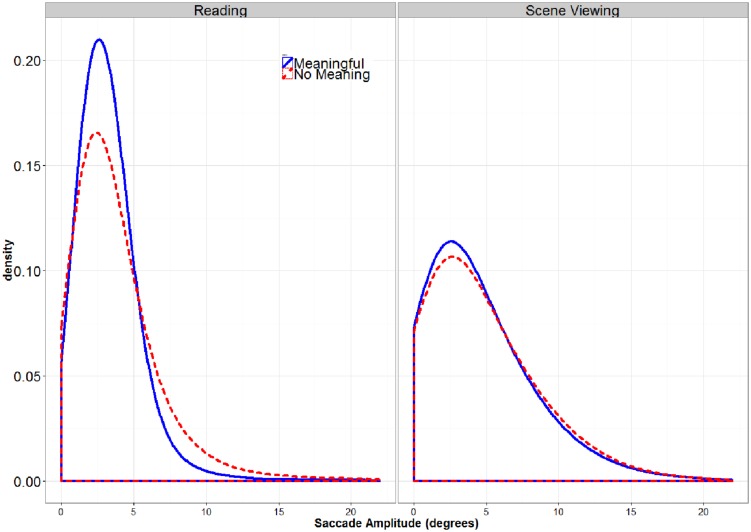
**Global Distribution of Saccade Amplitudes.** There was a greater proportion of very short and long saccades for the pseudo-stimuli (dashed lines) than for the meaningful stimuli, especially for the reading task.

A by-participant 2 (TASK: Reading vs. Scene Viewing) × 2 (STIMULUS TYPE: Meaningful vs. Pseudo-Stimulus) ANOVA was conducted on saccade amplitude. In this analysis, both main effects were significant, as was the interaction (all *F*s > 9.12, all *p*s < 0.0061). Follow-up *t*-tests showed that the significant interaction indicated that the effect of STIMULUS TYPE was present for reading but was not significant for scene viewing (Reading *t*(39) = -2.82, *p* = 0.0082, difference = 0.44; Scene Viewing *t*(39) = -0.63, *p* = 0.53, difference = 0.1). Interestingly, the mean saccade amplitude was shorter in reading than in pseudo-reading (see **Table 1**). This contradicts findings from previous studies using similar pseudo-reading tasks, where longer mean saccades were observed in normal reading than in pseudo-reading (Vitu et al., 1995; Rayner and Fischer, 1996; Luke and Henderson, 2013). Luke and Henderson (2013) observed that these mean differences were due to a greater proportion of very short and long fixations in pseudo-reading. **Figure 3** shows that the same pattern of results was obtained for reading in the current study. A similar increase in the proportion of longer saccades is observable for pseudo-scene viewing, although this shift was not large enough to significantly influence the means. Thus, although the findings of the present study with regard to mean saccade amplitudes may appear to contradict previous findings that saccades are shorter is pseudo-reading, the pattern of changes in the distribution of saccade amplitudes is the same.

### Fixation Duration

**FIGURE 4:**
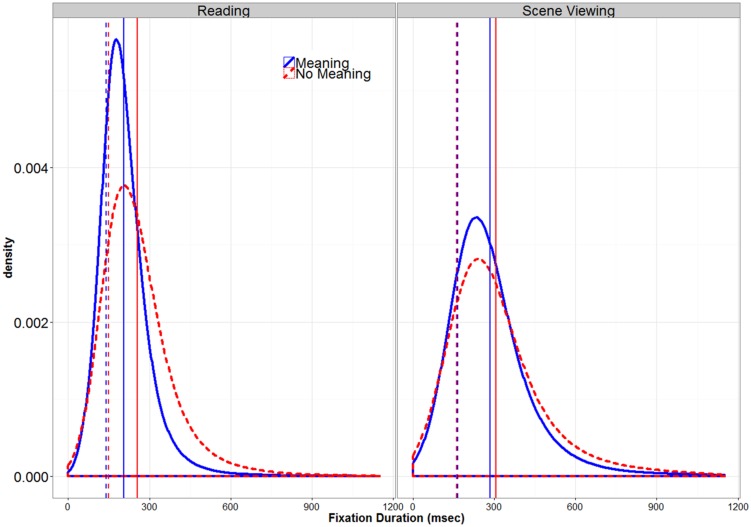
**Global Distribution of Fixation Durations.** The solid vertical lines represent overall means, while the dotted vertical lines represent the values for μ derived from the response time distribution analysis.

**Figure 4** shows the distribution of fixation durations for both scenes and text in the meaningful and pseudo-stimulus conditions. This figure illustrates that fixation duration distributions are not normal but are skewed to the right. Any difference in means between the meaningful and pseudo-stimuli might reflect a difference in the center of the two distributions, which occurs when most fixations in one condition are longer. However, since means are strongly influenced by outliers and extreme scores, a difference between means can also occur because one distribution is more skewed than another, which occurs when some subset (but not all) of the fixations are longer. Mean differences can reflect either of these differences or both in combination (Balota and Yap, 2011). Since the center and skew of fixation duration distributions vary independently of each other and often reflect different processes (Staub and Benatar, 2013), it is important to consider them separately.

To test whether the removal of meaningfulness had similar effects in the different tasks, the fixation duration distributions for each participant in each condition were analyzed using a response time distributional analysis (Balota and Yap, 2011). This analysis fits participants’ response time data with an ex-Gaussian distribution (Ratcliff, 1979), which is the convolution of a normal (Gaussian) distribution and an exponential distribution, with two parameters representing the normal component (μ, the mean, and σ, the SD), and a single exponential parameter (τ). Any changes in μ and σ indicate changes in the distribution’s normal component (i.e., the center), whereas increases in τ indicate increased skew to the right. Ex-Gaussian distributions fit eye-movement data quite well (Staub et al., 2010; Staub, 2011; White and Staub, 2012; Luke and Henderson, 2013; Luke et al., 2013). The ex-Gaussian distribution was fitted to the data from each participant in each task in each meaning condition using QMPE software (Heathcote et al., 2004). The mean ex-Gaussian parameters are found in **Table 2**.

**Table 2:** Parameters from the response time distributional analysis of fixation durations.

	Reading			Scene viewing		
		
	μ	σ	T	μ	σ	τ
Meaningful	139	38	66	164	52	122
Pseudo-stimulus	148	42	101	161	52	152
Effect	9	4	35	-3	0	30


*The three parameters μ, σ, and τ are from the response time distributional analysis of the fixation duration distributions shown in **Figure 3**. The first two parameters represent the normal component of the distribution (μ, the mean, and σ, the standard deviation), and τ is the exponential parameter representing the skew of the distribution. Mean log likelihoods for the four different conditions are: Reading, Meaningful: -2460.22; Reading, Pseudo-Stimulus: -2015.7; Scenes, Meaningful: -1345.4; Scenes, Pseudo-Stimulus: -1173.18.*

Previous research comparing fixation duration distributions in scene viewing and reading has observed that the distribution in scene viewing is both shifted to the right and more skewed to the right compared to reading (Luke et al. (2013); see also Henderson and Hollingworth (1998)). The removal of meaningfulness from a text stimulus has been shown to result in a “fatter tail”, skewing the distribution to the right compared to the normal, meaningful stimulus condition, but does not appear to influence μ or σ (Luke and Henderson, 2013). **Figure 4** and **Table 2** suggests that the primary difference between the Meaningful and Pseudo-Stimulus conditions in both tasks is indeed an increase in skew in the Pseudo-Stimulus condition, with no large shifts in the center of the distribution apparent. To look for any interactions between task and stimulus type that might indicate task-based differences in fixation duration control, especially in the analysis of the skew of the distribution (τ), by-participant 2 (TASK: Reading vs. Scene Viewing) × 2 (STIMULUS TYPE: Meaningful vs. Pseudo-Stimulus) ANOVAs were conducted on all three of the ex-Gaussian parameters. In the analyses of μ and σ, there were main effects of TASK (both *F*s > 43.75, all *p*s < 0.0046), indicating that both parameters were larger for scenes than for text. The main effects of STIMULUS TYPE were not significant (both *F*s < 3.9, both *p*s > 0.056). The interaction of the two factors was significant in both analyses (both *F*s > 6.9, all *p*s < 0.012), indicating that the effect of TASK was somewhat smaller in the Pseudo-Stimulus condition (Meaning: both *t*s > 5.61, both *p*s < 0.0001; No meaning: both *t*s > 2.28, both *p*s < 0.025). When the effect of STIMULUS TYPE was considered separately for each task, no significant differences were found in the analysis of μ (Reading: *t*(39) < 1.78, *p* = 0.072; Scene Viewing: *t*(39) < 0.57, *p* > 0.57). The effect was significant (although numerically tiny, only 4 ms) for reading only in the analysis of σ (*t*(39) < 2.03, *p* = 0.046; Scene Viewing: *t*(39) < 0.07, *p* > 0.94). These results indicate that if semantic content has an influence on the center or spread of the fixation duration distributions, such influences are quite small (<10 ms; see **Table 2**) and mostly non-significant. Accordingly, between-task differences in the influence of semantic content on μ or σ, if they exist at all, are on the order of a few milliseconds. Thus, these results are consistent with previous studies (Luke and Henderson, 2013).

In the analysis of τ, both main effects (TASK and STIMULUS TYPE) were significant (both *F*s > 99.06, both *p*s < 0.0001). The interaction of the two was not significant (*p* > 0.32). These findings indicate that while all three parameters of the fixation duration distribution were larger in scene viewing than in reading, consistent with previous research (Luke et al., 2013), meaningfulness only had a significant effect on the skew of the distribution (Luke and Henderson, 2013). The size of this effect was statistically the same in the two tasks.

## Discussion

The present study investigated how the meaningfulness of a visual stimulus influences how our eyes move. More specifically, we globally manipulated the meaningfulness of both texts and scenes in a within-subjects design, enabling us to explore potential differences in eye movement control across two visual tasks.

Saccade amplitudes were found to increase significantly in reading for pseudo-text. This difference in mean saccade amplitude across conditions appears to result from an increase in very short and long saccade amplitudes when meaning is removed from text. This shift in the distribution has been observed in other studies (Luke and Henderson, 2013), although in these studies means decreased because the proportion of short saccades increased more than was observed here. In scene viewing there was a numeric trend toward an increase (see **Table 1**) but it was small and far from significant. There was some suggestion of an increase in the proportion of longer fixations for pseudo-scenes as well. The absence of a significant effect of semantic content on saccades amplitudes in scene viewing may simply reflect a ceiling effect; saccades are larger by default in scene viewing, and it is probably not possible to increase them much more and still keep the eyes within the bounds of the stimulus. Regardless, these observed changes in saccade amplitude likely reflect a reduced need for foveal processing when the stimulus is not being processed for meaning.

One goal of the present study was to investigate whether the influence of stimulus meaningfulness differs across tasks. A close look at the distribution parameters from the fixation duration distribution analysis (**Table 2**) shows that the removal of meaningfulness affected the fixation duration distributions in the same way in both tasks, influencing the skew (τ) but not consistently influencing the center (μ, σ) of the distributions. That is, the distribution analysis showed no consistent evidence of any significant effects of meaning on either μ or σ. There was, however, a main effect of stimulus type in the analysis of τ. Further, the interaction of stimulus type and task was not significant in the analysis of τ, indicating that the removal of meaningfulness from the stimulus had a statistically identical influence in reading and in scene viewing. Thus, it appears that the influence of cognitive control on eye movements in reading and scene viewing is both qualitatively and quantitatively similar; not only did the removal of meaningfulness influence the same component of the distribution in both tasks, the magnitude of that influence was nearly identical. This observation is highly consistent with other research with the SOD paradigm showing that eye movements respond similarly to processing difficulty in reading and in scene viewing (Luke et al., 2013).

Increases in τ like those observed here occur when some, but not all, of the fixations are longer, which elongates the tail of the distribution but does not significantly shift its center. Thus, the fact that for the pseudo-stimuli τ was increased but the other parameters of the ex-Gaussian distribution were not indicates that not all fixations were affected by the removal of meaningfulness. This finding fits nicely with research using the SOD paradigm that reveals two populations of eye movements, providing converging evidence that longer duration fixations are under cognitive control and shorter duration fixations are not (Henderson and Pierce, 2008; Henderson and Smith, 2009; Nuthmann and Henderson, 2012; Luke et al., 2013). This finding is most consistent with models of eye movement control in which only longer fixations are influenced by the currently fixated stimulus (e.g., the competition-inhibition theory; Yang and McConkie (2001).

While the global manipulation employed here is useful for cross-task comparisons, it of course has certain limitations. Since our manipulation altered the stimuli in multiple ways, removing or changing some low-level visual features as well as obscuring the identity of words and objects, it is not possible to determine *which* cognitive processes (or which stage in processing) has the most influence on eye movements. This technique cannot therefore adjudicate cleanly between different proposals about the nature and source of cognitive control. The present study does, however, provide additional evidence, first, that reading, and scene viewing share a common control mechanism, and, second, that only some fixations are under the direct influence of the visual stimulus. Most models of eye-movement control apply to reading only (Reichle et al., 1998; Engbert et al., 2002, 2005), and so may not generalize to other tasks (but see Reichle et al. (2012) for an example of how E–Z reader can generalize to non-reading tasks). One model of eye-movement control that has been shown to successfully predict eye movements in both reading and scene viewing is the CRISP model (Nuthmann et al., 2010; Nuthmann and Henderson, 2012). CRISP also predicts that some eye movements will not be under cognitive control; fixation duration is determined by a random walk timer, after which a new saccade program is initiated. Prior to saccade program initiation, cognitive intervention can occur via inhibition of the saccade program when processing difficulty is encountered, but after the timer expires no cognitive intervention is possible. Thus, CRISP is consistent with the findings of this and other studies. The results of the current study suggest that current and future models of eye movement control should, first, be able to account for eye movements across multiple tasks, and second, incorporate a mechanism for cognitive control that exempts some subset of fixations.

## Author Contributions

Both authors (SL, JH) made substantial contributions to the conception or design of the work. SL was primarly responsible for the acquisition, analysis, or interpretation of data for the work. SL Drafted the work and JH revised it critically for important intellectual content. Both SL and JH gave final approval of the version to be published, and agree to be accountable for all aspects of the work in ensuring that questions related to the accuracy or integrity of any part of the work are appropriately investigated and resolved.

## Conflict of Interest Statement

The authors declare that the research was conducted in the absence of any commercial or financial relationships that could be construed as a potential conflict of interest.
